# Diverse Diets with Consistent Core Microbiome in Wild Bee Pollen Provisions

**DOI:** 10.3390/insects11080499

**Published:** 2020-08-04

**Authors:** Rebecca M. Dew, Quinn S. McFrederick, Sandra M. Rehan

**Affiliations:** 1 Department of Biology, York University, Toronto, ON M3J 1P3, Canada; rebecca.dew11@gmail.com; 2 Department of Entomology, University of California Riverside, Riverside, CA 92521, USA; quinnmc@ucr.edu

**Keywords:** pollination, microbiome, foraging, bees, *Ceratina*, bacteria

## Abstract

Bees collect pollen from flowers for their offspring, and by doing so contribute critical pollination services for our crops and ecosystems. Unlike many managed bee species, wild bees are thought to obtain much of their microbiome from the environment. However, we know surprisingly little about what plant species bees visit and the microbes associated with the collected pollen. Here, we addressed the hypothesis that the pollen and microbial components of bee diets would change across the range of the bee, by amplicon sequencing pollen provisions of a widespread small carpenter bee, *Ceratina calcarata*, across three populations. *Ceratina calcarata* was found to use a diversity of floral resources across its range, but the bacterial genera associated with pollen provisions were very consistent. *Acinetobacter*, *Erwinia*, *Lactobacillus*, *Sodalis*, *Sphingomonas* and *Wolbachia* were among the top ten bacterial genera across all sites. *Ceratina calcarata* uses both raspberry (*Rubus*) and sumac (*Rhus*) stems as nesting substrates, however nests within these plants showed no preference for host plant pollen. Significant correlations in plant and bacterial co-occurrence differed between sites, indicating that many of the most common bacterial genera have either regional or transitory floral associations. This range-wide study suggests microbes present in brood provisions are conserved within a bee species, rather than mediated by climate or pollen composition. Moving forward, this has important implications for how these core bacteria affect larval health and whether these functions vary across space and diet. These data increase our understanding of how pollinators interact with and adjust to their changing environment.

## 1. Introduction

The broader community of microbes within a host, the microbiome, can determine the health status of an individual. Many microbes provide beneficial functions for the host including metabolism and immunity [1,2]. In honey bees, certain *Lactobacillus* strains offered protection against a microsporidian and bacterial pathogen [3]. Similarly, in bumble bees, increased microbiome diversity was linked to reduced infection by the trypanosomatid parasite *Crithidia* [4]. In *Osmia ribofloris*, the pollen provision microbiome is crucial for larval development [5]. Therefore, it is important to characterize and understand the microbiome to understand bee health.

Our current knowledge on the microbiome of bees is predominantly based on honey bees (*Apis* spp.), and to a lesser degree bumble bees (*Bombus* spp.) [6,7,8]. Both of these are highly social and closely related members of the corbiculate apid bees, and as such they share a very similar core microbiome [7]. Outside of these genera, the bee microbiomes sequenced so far do not conform to the *Apis* and *Bombus* models. Even within the corbiculates, the stingless bees (Meliponini) and the orchid bees (Euglossini) lack some of the most common symbionts of *Apis* and *Bombus*, although several related symbionts are shared amongst the corbiculates [7]. Looking more broadly, bacteria that were previously classified as *Lactobacillus* but have been recently split into the genera *Apilactobacillus, Bombilactobacillus* and *Lactobacillus* sensu strictu [9] are some of the few symbionts common to multiple bee taxa including *Apis*, *Bombus*, the small carpenter bee *Ceratina,* megachilid and halictid bees [10,11,12,13,14,15]. Microbe acquisition in *Apis* and *Bombus* occurs within the hive, facilitated by nestmate interactions or transfer from feces [6,12,16,17,18]. While honey and bumble bees live in large groups, this level of sociality is rare among bees, the vast majority of bee species being solitary [19]. Indeed, in the other bee species studied so far, much of their microbiota appears to be gained from the environment rather than through social transmission [11,13,14,20,21,22]. Therefore, differences in environmental and pollen-associated bacteria may have larger impacts on wild bee development than for the highly social corbiculate bee species.

Microbial acquisition from the environment may be influenced by the diet of the bee. As for bees, flowers harbor a variety of microbes, which can potentially be passed to foraging bees. *Crithidia* can be transmitted between foraging *Bombus* at flowers [23] and communities of pollinators have been found to share microbes [24,25]. For bees that use foliage to line their nests, both flower and foliar source affect their pollen provision microbiome [22]. However, there are also many more complex factors to consider such as flower morphology, volatiles and even the secondary compounds produced by the microbes themselves, that can alter floral bacterial communities and transmission to pollinators [26,27]. Therefore, diet may be an important factor to consider when looking at the wild bee microbiome, which is thought to be largely environmentally sourced.

To conserve wild bees, we need to understand their health, and their associated microbial symbionts. It seems likely that the microbes present in the environment, and therefore those gained environmentally by bees, will vary geographically with changes in climate, interacting insect species and floral communities.

*Ceratina calcarata* Robertson, 1900 is a small carpenter bee species that is a widespread and prominent pollinator across eastern North America [28,29,30]. This species nests in the dead stems of various plants, commonly raspberry and sumac (*Rubus* and *Rhus* species, respectively) [20,31]. The plants it nests in also produce flowers, potentially biasing pollen collection and thereby microbial acquisition. This bee constructs separate brood cells within the stem nest, each provisioned with a single pollen ball on which an egg is laid [31]. This brood provision is the only source of food given to the offspring until it reaches maturity. Study of these brood provisions from nests at the northern extent of its range found they contain multiple pollen species and a diversity of microbes dominated by *Lactobacillus*, *Wolbachia*, *Acinetobacter* and *Sodalis* [20]. However, *C. calcarata* is found across a broad geographic range in eastern North America and acquires at least part of its microbiome from the environment [20], therefore its microbiome may vary geographically with corresponding changes in climate and floral landscape.

The aim of this project was to investigate whether the microbiome of *C. calcarata* varies geographically by sequencing brood provisions spanning this species’ range across the eastern United States. Specifically, we asked whether pollen or bacterial species vary in composition or diversity among sites and if there are identifiable plant/microbe associations. We also asked whether foraging was biased by the proximity of nest plant flowers.

## 2. Methods

### 2.1. Brood Provision Collection

**Figure 1:**
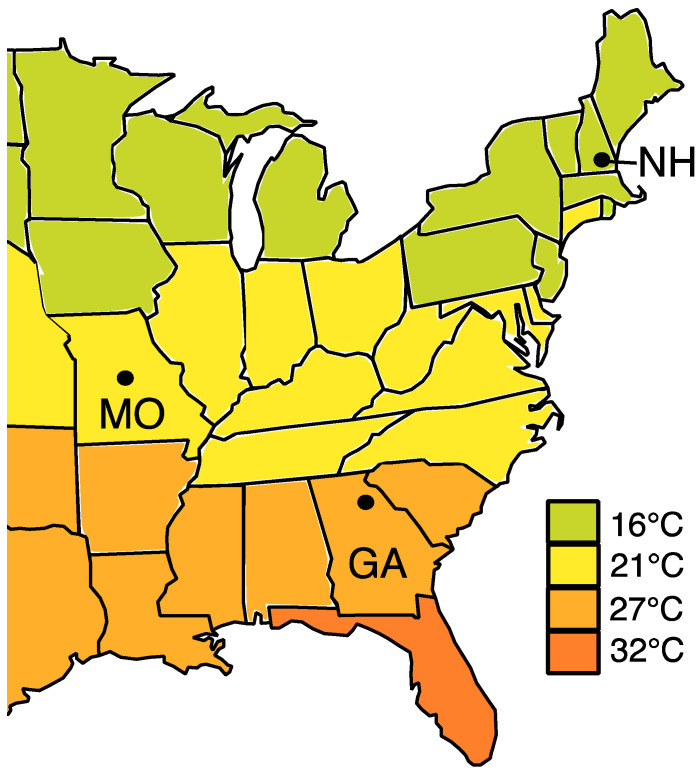
Map showing the average summer temperatures (°C) across the eastern United Sates. Black dots indicate the sampled regions of GA: Georgia, MO: Missouri and NH: New Hampshire. Map was modified from the National Oceanic and Atmospheric Administration (http://www.noaa.gov/climate).

Nests of *Ceratina calcarata* were collected from Athens, Georgia (33.95’19”° N, 83.35’76”° W), Lake Ozark, Missouri (38.19’86”° N, 92.63’88”° W) and Durham, New Hampshire, USA (43.13’39”° N, 70.92’64”° W). These sites cover the geographic and climatic range of this bee within the USA (Figure 1). Nests were collected in dead stems of *Rubus* spp. (raspberry) and *Rhus* spp. (sumac) from May to July 2016. The stems were split open and brood provisions from each nest were removed with flame-sterilized forceps and placed into separate cryovials. These were stored at −80 °C prior to DNA extraction.

### 2.2. DNA Extraction and Sequencing

DNA extraction was performed with the DNeasy blood and tissue kit (Qiagen, Valencia, CA, USA). Each sample was homogenized with a sterile steel bead (5 mm) and 100 μL of sterile glass beads (0.1 mm) in 180 μL ATL buffer using a Qiagen tissue lyser. An aliquot of 20 μL proteinase K was added to the homogenized samples, which was then incubated overnight at 56 °C. Three negative controls containing sterile water were simultaneously prepared.

PCR reactions targeted the 16S ribosomal RNA gene to sequence bacterial species, and the ribulose bisphosphate carboxylase large subunit (rbcl) to sequence pollen species. Primers and PCR assays for both genes followed the protocols outlined by McFrederick and Rehan [20]. The products of the PCR were cleaned using the PureLink Pro Purification Kit (Invitrogen, Carlsbad, CA, USA). Then, a second PCR was performed with 1 μL of each of the cleaned PCR1 products as template to add the Illumina adapters. The PCR2 products were then normalized by running 18 μL of the product through SequelPrep normalization plates (Invitrogen, Carlsbad, CA, USA). The pooled normalized product was cleaned a second time via a speed bead cleaning method before the libraries were quality checked with a 2100 Bioanalyser (Agilent, Santa Clara, CA, USA). Sequencing was performed with the MiSeq Reagent Kit v3, using 2 × 300 cycles. The raw sequences can be accessed on the NCBI Sequence Read Archive (SRA) under accession number PRJNA454884.

## 3. Data Analysis

The data for both 16S and rbcl were demultiplexed in Qiime2 v.2017.10 [32]. Quality filtering and exact sequence variant (ESV) clustering were performed with the Qiime2 plug-in Dada2 [33]. The 16S rRNA gene reads were classified in Qiime2 using a naïve Bayes classifier trained on the Greengenes 99% database [34]. The resulting taxonomy was filtered to remove all mitochondrial and chloroplast genes. The insect symbionts *Wolbachia* and *Sodalis* were recovered in reads. It is unlikely these were obtained from flowers, more likely coming from the mother or mites in the nest. We chose to keep these reads, as we do not know the source of any of the bacteria found in the pollen provision, or whether these are metabolically active, only that they are present. In this way, we give a complete snapshot of the community present.

**Figure 2:**
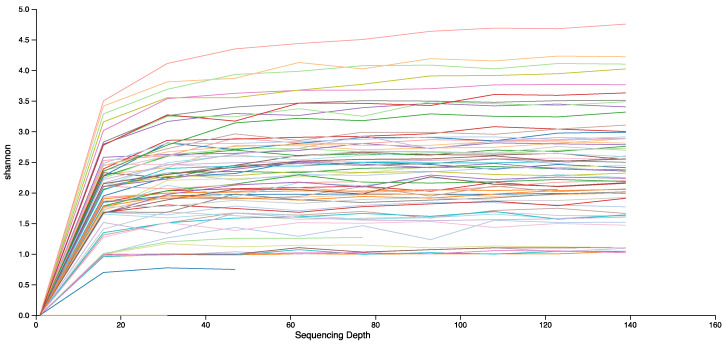
Rarefaction curve of Shannon diversity for the 16S reads for each sample to a read depth of 139 reads. Based on the dataset with the *Sodalis* reads removed.

There were some bacterial reads in one of the negative controls. Most of these were common laboratory contaminants and were removed from all reads [35]. However, *Sodalis* was found in 6.140% of the control reads. *Sodalis* has been previously identified in brood provisions of *C. calcarata* [20], and was present in 40 of our brood provision samples, with an average of 8.61% reads per sample. Given the high frequency of *Sodalis* in some samples, it is likely to represent a true component of the microbial community in the brood provisions. To compensate for this, we analysed the data in two separate ways, (i) removing all *Sodalis* reads from samples, and (ii) removing a portion of *Sodalis* from each sample, up to a maximum of 6.140% of the sample’s reads, to correspond to the percentage of contamination present in the control. We did not use this adjusted ratio of *Sodalis* in analyses of abundance, as this is reliant on accurate read counts. Alpha and beta diversity analyses were performed on both of these datasets at a read depth of 139 reads, allowing inclusion of 87% and 89% of samples in each dataset, respectively. Read depth was shallow, as the quality and filtering steps removed a large proportion of reads, but the rarefaction curves plateaued at this depth, indicating that bacterial diversity was truly low in these samples (Figure 2).

For rbcl, read quality was low for all New Hampshire sequences, so these were not analysed further. However, *C. calcarata* pollen balls collected from New Hampshire in July 2014 were previously sequenced for rbcl by McFrederick and Rehan [20]. In that study, the sequences were analysed in Qiime1 using 97% OTU matching. This software has now been superseded by Qiime2, which employs exact sequence variant matching. As part of this change, the Qiime2 program also implements more stringent quality filtering. Here, we reanalyse the rbcl data from McFrederick and Rehan [20] in Qiime2, to allow for statistical comparison to the rbcl data generated in the current study for Missouri and Georgia. The New Hampshire reads went through demultiplexing, quality filtering and ESV binning separately, to account for sequencing run differences before amalgamation with the Georgia and Missouri rbcl reads.

The rbcl ESVs from Georgia, Missouri and New Hampshire that remained after quality filtering were assigned taxonomy based on local BLAST searches with 99% sequence identity [36]. All plant genera returned by BLAST searches were confirmed to be in Georgia and Missouri through searches in the USDA PLANTS Database (http://plants.usda.gov/checklist.html) and the Missouri Botanical Gardens Plant Finder (http://www.missouribotanicalgarden.org). Diversity analyses were performed at a depth of 3791 reads, preserving 79% of samples.

We considered Faith’s phylogenetic diversity as a measure of taxonomic richness; comparisons between groups were made with Kruskal–Wallis analysis (K–W). Abundance data were compared through PERMANOVA analysis of both Bray–Curtis dissimilarity (B-C) and weighted unifrac (W-U) indices as measures of beta-diversity and phylogenetic beta-diversity, respectively. Additional statistical comparisons were performed with Kruskal–Wallis and Wilcoxon tests in R v.1.0.136.

Co-associations between the pollen and bacterial components of the brood provisions were assessed using SparCC [37] and CoNet [38]. Both programs were used as SparCC and CoNet use correlation and dissimilarity methods, respectively, and the differences in the algorithms between these two approaches can lead to different results [39]. New Hampshire was not considered due to the exclusion of the rbcl data for this site, and these analyses have been previously reported for New Hampshire in [20]. Analyses were run on a combined dataset of all bacterial and plant ESVs identified to genus level from Georgia and Missouri. This was then repeated on separate datasets of reads from Georgia and Missouri separately to look for site differences in co-associations. Pseudo p-values in SparCC were calculated based on 100 bootstrap replicates. In CoNet, network edge scores were calculated with Pearson, Spearman, mutual information, Bray Curtis and Kullback Leibler. Bootstraps were calculated using Brown p-value merging and Benjamini–Hochberg multiple test correction. Positive correlations were only considered if recovered in both SparCC and CoNet with *p* ≤ 0.01.

## 4. Results

After trimming, quality filtering and chimera removal, 350,472 sequences of 16S were obtained for 189 ESVs across 65 samples. This dropped to 250,810 reads in 62 samples when *Sodalis* was excluded due to its presence in the negative controls. There were 299,489 rbcl sequences for 249 ESVs across 79 samples from Missouri and Georgia, with a total of 44 samples with sequences for both gene regions. For the New Hampshire rbcl data reanalysed from [20], there were 356,354 rbcl sequences for 226 ESVs across 94 samples.

The Missouri and New Hampshire pollen rbcl samples were exclusively from nests formed in sumac plants, but the Georgia samples comprised 35 nests in raspberry and 22 nests in sumac (Table S1). The 62 samples successfully sequenced for 16S included 24 nests from Missouri, 31 from Georgia and 7 from New Hampshire. Nests from Missouri were exclusively from sumac, and New Hampshire nests were from raspberry (n = 5) and sumac (n = 2), as were the nests from Georgia (raspberry: n = 17; sumac: n = 14; Tables S2 and S3).

**Figure 3:**
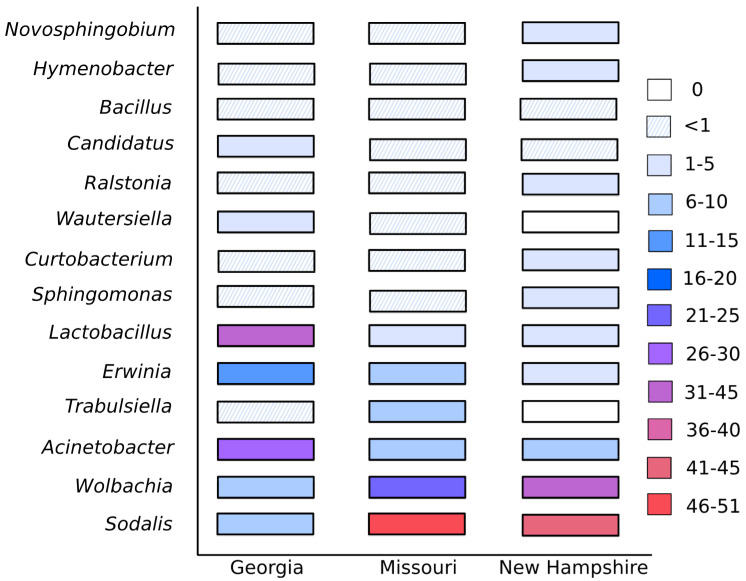
The percentage of 16S reads found across the top 14 bacterial genera in brood provisions from each site. Many bacteria were common to all sites but the relative abundance of the most common bacteria varied broadly.

In total, 58 bacterial ESVs were classified through to genus, however the provisions were dominated by just a few of these genera. In each site, > 96% of the sample reads were from the 10 most common genera. *Acinetobacter*, *Erwinia*, *Lactobacillus*, *Sodalis*, *Sphingomonas* and *Wolbachia* were found among those top 10 at all sites. For the analyses using an adjusted percentage of *Sodalis*, this was the most frequent genus in Missouri, covering 51% of the reads (Figure 3). The second most common genus at that site was *Wolbachia*. For Georgia, *Lactobacillus* was the top genus, having 32% of the reads (Figure 3). There were very few bacterial genera unique to each state: Georgia had nine unique genera, Missouri five and New Hampshire one. Consequently, phylogenetic microbial richness did not significantly differ among states (K–W: N_GA_ = 31, N_MO_ = 27, N_NH_ = 7, H = 3.57, P = 0.167) or with nesting substrate (N_R_ = 22, N_S_ = 40, H = 0.966, P = 0.318). The results were the same if *Sodalis* was excluded (states: N_GA_ = 31, N_MO_ = 24, N_NH_ = 7, H = 0.867, P = 0.684; nesting substrate: N_R_ = 22, N _S_ = 40, H = 0.146, P = 0.702). However, when abundance was considered in the analyses excluding *Sodalis*, the microbial composition of the brood provisions did significantly differ between Georgia nests in raspberry and the Missouri nests, all of which were in sumac (B–C: pseudo-F_41_ = 4.63, q = 0.010, W-U: pseudo-F_41_ = 6.23, q = 0.040). There was also a significant difference in phylogenetic diversity between Georgia nests in sumac and Missouri nests, though this was not significant for non-phylogenetic diversity (W-U: pseudo-F_38_ = 4.45, q = 0.045; B–C: pseudo-F_38_ = 2.90, q = 0.065). The bacterial composition of brood provisions did not significantly differ between all other pairwise comparisons, including comparisons of Georgia and Missouri with New Hampshire.

**Figure 4:**
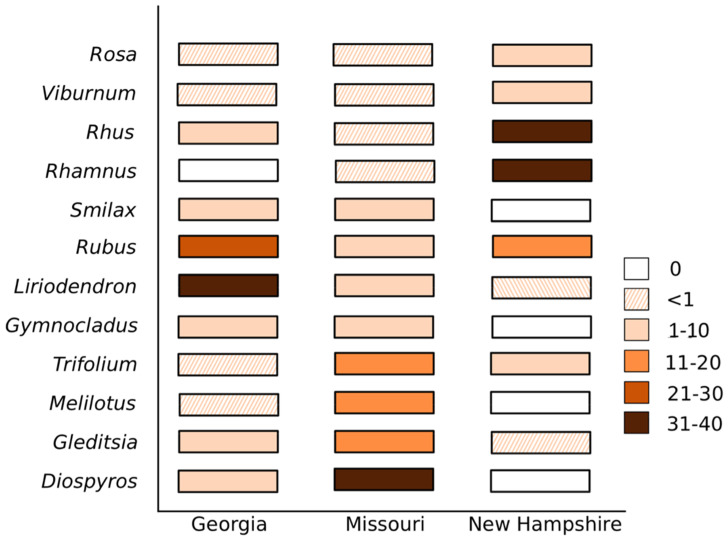
The percentage of rbcl reads found across the top 12 plant genera in brood provisions from each site. The most abundant plant genera varied between each state.

There were in total 96 plant genera present in the brood provisions. Brood provisions in Georgia contained 52 plant genera, with *Liriodendron tulipfera* and *Rubus spp.* accounting for 31% and 28% of the reads, respectively (Figure 4). Missouri had 40 genera dominated by *Diospyros* (38%) and *Gleditsia* (15%; Figure 4). New Hampshire had 65 genera, predominantly consisting of *Rhamnus* (36%) and *Rhus* (32%). Phylogenetic richness was significantly higher in New Hampshire (mean = 0.44, s.e. = 0.23) when compared to Georgia (mean = 0.41, s.e. = 0.31; K–W: N_NH_ = 94, N_GA_ = 57, H = 6.38, q = 0.035). Neither Georgia nor New Hampshire significantly differed in richness from Missouri (mean = 0.40, s.e. = 0.29; K–W: N_NH_ = 94, N_MO_ = 22, H = 1.64, q = 0.300; N_GA_ = 57, N_MO_ = 22, H = 0.62, P = 0.431). There was also no significant difference in phylogenetic richness with nesting substrate (K–W: N_RUBUS_ = 35, N_RHUS_ = 138, H = 0.91, q = 0.341). However, the number of pollen genera in each individual brood provision was higher in Missouri (mean: 10.24 ± 1.3 s.e.) compared to Georgia (mean: 6.67 ± 0.5 s.e.; pairwise Wilcoxon, Bonferroni correction: P = 0.024), while the number of pollen genera in New Hampshire provisions did not significantly differ from either Missouri (P = 0.305) or Georgia (P = 0.225). 

Brood provisions significantly differed in pollen beta-diversity between Missouri and New Hampshire (B–C: pseudo-F_116_ = 21.53, q = 0.001; W-U: pseudo-F_116_ = 32.59, q = 0.001), Missouri and Georgia (B–C: pseudo-F_79_ = 12.35, q = 0.001; W-U: pseudo-F_79_ = 12.87, q = 0.001) and New Hampshire and Georgia (B–C: pseudo-F_151_, q = 0.001; W-U: pseudo-F_151_ = 35.25, q = 0.001). No significant differences in beta-diversity were found between *Rubus* and *Rhus* nests within Georgia (B–C: pseudo-F_57_ = 0.82, q = 0.517, W-U: pseudo-F_57_ = 0.34, q = 0.742). To test if pollen of nest plants is preferentially collected, we compared the number of *Rubus* reads between nests formed in *Rubus* or *Rhus* stems in Georgia, and we found no significant difference (Wilcoxon: W = 421.5, *p* = 0.555). There was also no significant difference in the amount of *Rhus* pollen collected (Wilcoxon: W = 365, *p* = 0.680).

Significant positive correlations between plant and bacterial genera were not consistent between the analysis of the overall dataset and state-level analyses (Table 1). In the overall analysis of Georgia and Missouri combined, a significant correlation was found between the plant genus *Liriodendron* and *Lactobacillus*. Analysis of Georgia separately gave a significant correlation between *Liriodendron* and *Sphingomonas*. Within the Missouri data, a number of significant correlations were found, though none involving *Liriodendron*. For Missouri, the plant genera *Brunia, Camptotheca, Rhus* and *Smilax* were all correlated with *Wolbachia*. The plants *Gleditisia* and *Gymnocladus* were correlated with *Trabulsiella*, while *Trifolium* was correlated with *Wautersiella*. In New Hampshire, based on data presented in [20], correlations were found between *Gleditsia* and *Rosa* with *Lactobacillus* and *Rubus* was correlated with *Acinetobacter* and *Sodalis* (Table 1).

## 5. Discussion and Conclusions

The floral resources utilized by *C. calcarata* differed between regions, brood provisions being dominated by different pollen genera in each state, showing this generalist bee’s local adaptation to regional floral communities (Figure 4). Foraging preference was not biased towards flowers present on the host nest plant, indicating that spatial assortment of floral resources alone does not determine foraging preferences. Despite changes in floral resources, the same core microbes dominated brood provisions across all states, although the relative abundance of these groups did vary between region (Figure 3). Our overall network analyses identified some correlations in plant and bacterial occurrences, however the broad changes in floral diet between states did not correspond to large changes in the bacterial community, suggesting that these floral–bacterial associations are transient or non-obligate (Table 1).

There were a number of core bacterial genera found across all sites but the relative abundance of these varied strongly (Figure 3). Comparison to McFrederick and Rehan’s [20] study in New Hampshire suggests there may also be annual variation within sites. In 2016, *Lactobacillus* was the most common genus, while we recovered *Sodalis* and *Wolbachia* as the top two genera in the current study (Figure 3). Being obligate facultative endosymbionts in many insect species, the occurrence of *Wolbachia* and *Sodalis* is likely due to contamination from mites or other parasites, or transfer directly from the mother’s crop rather than through any specific floral sources [40,41]. A major limitation of amplicon sequencing studies is that they can only determine the presence or absence of an organism’s DNA, not whether that organism is metabolically active, and this holds true for all bacteria recovered in our samples. This aside, it is interesting that *Wolbachia* contamination is so prevalent, and future microscopic examination of pollen material for mite infestation and tests of possible vertical transmission via pollen are needed. *Lactobacillus* was only present in 2.2% of reads in the New Hampshire samples for this study. Within Georgia, the abundance of bacterial ESVs differed between nests in *Rubus* and *Rhus* but significant differences disappeared when the phylogenetic similarity of bacteria was considered. This suggests there could be some differences in the abundance of microbial species or strains between nesting substrates but overall the taxonomic distribution of bacteria in the brood provisions is similar. Nesting substrate, therefore, has a smaller or negligible effect on bacterial abundance compared to differences among states.

*Ceratina calcarata* foraged from a slightly greater phylogenetic richness of floral genera in New Hampshire than in Georgia. The phylogenetic richness of floral genera in Missouri did not significantly differ from either Georgia or New Hampshire, perhaps because of its mid-lying geographic position and climate. As expected, our reanalysis of the New Hampshire data with 99% ESV matching recovered fewer genera than in McFrederick and Rehan [20], who found 110 genera compared to this study with 65. The genera not recovered in our reanalysis were all present in less than 1% of reads in the original study. We identified the same five genera as being the most abundant (*Rhamnus*, *Rhus*, *Rubus*, *Viburnum*, *Trifolium*), these genera accounting for 92% of the reads (Figure 4). Despite this more conserved estimate of genera, the floral resources used in New Hampshire are still rich compared to those utilized in Georgia. We also found that foraging females in Missouri foraged from more plants to form a single pollen provision mass than those in Georgia (on average 10.2 genera for females in Missouri compared to 6.7 for females in Georgia). This suggests that suitable floral resources at the time of brood provisioning may not be as diverse in Georgia as more northern areas of *Ceratina*’s range, or that they were simply not locally abundant in the area around the collected nests.

Across its geographic range, *C. calcarata* encounters a broad variety of possible forage. Diets in Georgia, Missouri and New Hampshire were dominated by pollen from different plant genera (Figure 4). Out of the 96 floral genera found in provisions in this study, only *Rubus* was found in more than 1% of reads across all three states (Figure 4). All other genera, even if abundant in one or two states, were rare in provisions from the third. For example, sumac was a key floral resource in New Hampshire but made up less than 10% of the reads in Georgia and was hardly utilized at all in Missouri (<1% of reads; Figure 4). It is important to note that while read counts have been correlated with microscopy pollen counts in many studies [27,42,43,44], factors such as pollen morphology can skew the abundance estimate obtained from DNA sequences [45]. Our study uses the marker rbcl, which has shown strong correlation with pollen counts, outperforming *trnL* and ITS2 [27]. With this in mind, comparison of relative abundance between sites shows state-wise differences in diet. Many of these plant genera are common to all three states, so perhaps these dietary variations are due to differences in bee and floral phenologies, as well as possible microhabitat distinctions in floral assemblages in proximity to the bee nest.

While we do not have data on floral distributions within each collecting site, our records of nest substrate allow us to determine that foraging was not skewed towards the host plants. *Rubus* was a common pollen source but even nests formed within *Rubus* plants did not show a bias in pollen collection. Different pollens vary in nutritional qualities, which may influence foraging decisions [46]. Pollen can also have toxic constituents, and some generalist foragers appear to actively utilize a broad range of floral resources to alleviate the effects these may have on brood development [47,48]. How these factors influence *C. calcarata* foraging is unknown but our results suggest that spatial orientation of floral resources alone does not determine foraging preferences.

The presence of a consistent core microbial community despite the variation in pollen sources suggests that many of the most common bacterial genera do not have specific floral associations. We identified a number of tentative bacteria–plant correlations, but these were not consistent among states (Table 1). In the overall analysis, the tupliptree genus *Liriodendron* was correlated with *Lactobacillus*, while the same plant genus was correlated with *Sphingomonas* in Georgia. In Missouri, *Wolbachia* was correlated with four plant genera: *Brunia*, *Camptotheca*, *Rhus* and *Smilax* but this bacterium was not correlated with plants in the other states or the overall analysis. The correlations found in Georgia and Missouri also differ to those previously identified in New Hampshire, following the same methodology [20]. These correlations broadly suggest that plants and bacteria are co-occurring but the variance in results between the overall dataset and the state-level analyses indicates these relationships are facultative or transient. Using read data to identify co-occurrence correlations is statistically challenging [39,49] and further experiments sampling pollen bacterial communities with and without pollinator visitation, such as the study by McFrederick et al. [13], are needed to directly test for plant–bacteria associations.

Whether plants harbor certain microbes over others or not, there are many factors altering microbial floral communities. Long-term artificial warming of grassland plots was found to alter the microbial communities of plant leaves, including microbial groups common to bees [50]. Aydogan et al. [50] found *Acinetobacter* and *Wolbachia* increased in frequency, while *Sphingomonas* frequency decreased, these three bacterial genera being common to *C. calcarata* pollen provision and adult gut microbiomes [10,20]. These temperature based microbial changes could translate into changes in insect microbiomes, and indeed climate has been correlated with changes in microbiome composition in some species such as the red palm weevil [51], the chestnut weevil [52] and a spider mite [53]. Flower visitation by bees can transfer microbes to flowers, but herbivorous insects, other pollinators including thrips and wind are thought to contribute to microbe dispersal as well [13,54,55,56]. Similarly, the presence of potentially predatory or competitive species such as ants can reduce floral visitation and this in turn alters the microbes present on flowers [57]. Any and all of these could be important factors influencing the observed microbiome variation in *C. calcarata* and are important considerations when concerned with wild bee health generally.

Our study shows that the diet of *C. calcarata* varies widely with geography, with only *Rubus* found in more than 1% of reads at all three sites, indicating that this generalist bee species is able to utilize different resources as floral communities change. However, it seems that floral preference may not be simply determined by the proximity of the floral resource to the nest. The same six bacterial genera consistently dominated provisions in all sites but the relative abundance of these fluctuated widely. There are still many unknowns regarding how microbes are acquired, both in the pollen provisions and subsequently the bees themselves. Flowers appear to be general points of bacterial transmission, but so far specific associations have not been identified. The current lack of knowledge on microbial associates is a major hindrance in our ability to maintain diverse wild bee populations.

## Figures and Tables

**Table 1:** Correlations coefficients between plant and bacteria genera as identified by SparCC. Only significant positive correlations are shown that were also found to be significant in CoNet (*p* ≤ 0.01). Superscripts notate significant correlations found when analyses were restricted to Georgia (^GA^), Missouri (^MO^) or New Hampshire (^NH^).

Bacteria	Plants									
	*Brunia*	*Camptotheca*	*Gleditsia*	*Gymonocladus*	*Liriodendron*	*Rhus*	*Rosa*	*Rubus*	*Smilax*	*Trifolium*
*Acinetobacter*								0.287 ^NH^		
*Lactobacillus*			0.231 ^NH^		0.393		0.225 ^NH^			
*Sodalis*								0.249 ^NH^		
*Sphingomonas*					0.420 ^GA^					
*Trabulsiella*			0.579 ^MO^	0.578 ^MO^						
*Wautersiella*										0.654 ^MO^
*Wolbachia*	0.600 ^MO^	0.587 ^MO^				0.574 ^MO^			0.566 ^MO^	

## Supplementary Materials

The following are available online at https://www.mdpi.com/2075-4450/11/8/499/s1, Table S1: Rbcl reads for each sample binned by genus, Table S2: 16S reads with the number of *Sodalis* reads reduced by 6.140%, Table S3: 16S reads with all *Sodalis* reads removed.

## Data Accessibility

NCBI Sequence Read Archive (SRA) accession number PRJNA454884.
